# Air pollution and airway resistance at age 8 years – the PIAMA birth cohort study

**DOI:** 10.1186/s12940-018-0407-9

**Published:** 2018-07-17

**Authors:** Isabelle Finke, Johan C. de Jongste, Henriette A. Smit, Alet H. Wijga, Gerard H. Koppelman, Judith Vonk, Bert Brunekreef, Ulrike Gehring

**Affiliations:** 1 Institute for Risk Assessment Sciences, Utrecht University, P.O. Box 80178, 3508TD Utrecht, The Netherlands; 2 Department of Pediatrics, Division of Respiratory Medicine, Erasmus University Medical Center/Sophia Children’s Hospital, Rotterdam, The Netherlands; 3 Julius Center for Health Sciences and Primary Care, University Medical Center Utrecht, Utrecht, The Netherlands; 4 Center for Nutrition, Prevention and Health Services, National Institute of Public Health and the Environment, Bilthoven, The Netherlands; 5 Department of Pediatric Pulmonology, Beatrix Children’s Hospital, University Medical Center Groningen, University of Groningen, Groningen, The Netherlands; 6 Groningen Research Institute for Asthma and COPD, University of Groningen, Groningen, The Netherlands; 7 Department of Epidemiology, University Medical Center Groningen, University of Groningen, Groningen, The Netherlands

**Keywords:** Air pollution, Children, Interrupter resistance, Particulate matter, Nitrogen dioxide

## Abstract

**Background:**

Air pollution has been found to adversely affect children’s lung function. Forced expiratory volume in 1 s and forced vital capacity from spirometry have been studied most frequently, but measurements of airway resistance may provide additional information. We assessed associations of long-term air pollution exposure with airway resistance.

**Methods:**

We measured airway resistance at age 8 with the interrupter resistance technique (R_int_) in participants of the Dutch PIAMA birth cohort study. We linked R_int_ with estimated annual average air pollution concentrations [nitrogen oxides (NO_2_, NO_x_), PM_2.5_ absorbance (“soot”), and particulate matter < 2.5 μm (PM_2.5_), < 10 μm (PM_10_) and 2.5–10 μm (PM_coarse_)] at the birth address and current home address (*n* = 983). Associations between air pollution exposure and interrupter resistance (R_int_) were assessed using multiple linear regression adjusting for potential confounders.

**Results:**

We found that higher levels of NO_2_ at the current address were associated with higher R_int_ [adj. mean difference (95% confidence interval) per interquartile range increase in NO_2_: 0.018 (0.001, 0.035) kPa·s·L^− 1^]. Similar trends were observed for the other pollutants, except, PM_10_. No association was found between R_int_ and exposure at the birth address.

**Conclusions:**

Our results support the hypothesis that air pollution exposure is associated with a lower lung function in schoolchildren.

**Electronic supplementary material:**

The online version of this article (10.1186/s12940-018-0407-9) contains supplementary material, which is available to authorized users.

## Background

Lung development starts in utero and tracks throughout life [1, 2]. Therefore, maximum attained lung function in early adulthood likely will be suboptimal in those with a low lung function in early childhood and the threshold for respiratory symptoms and disability like chronic obstructive pulmonary disease will be reached earlier [3, 4].

There is growing evidence for adverse effects of long-term exposure to ambient air pollution on the lung function of children from cross-sectional and longitudinal studies [5–7]. Spirometry is considered the gold standard for measuring lung function and forced expiratory volume in 1 s (FEV_1_), is often used as a measure of airway obstruction in epidemiological studies [5]. However, reproducible spirometry is often not possible in children. Interrupter resistance (R_int_) requires less skill and cooperation, and is feasible in young children [8, 9]. Moreover, since air flow limitations are partly caused by increased airway resistance, direct measurements of airway resistance may provide additional information [10].

Only four studies so far investigated associations between long-term air pollution exposure and airway resistance and only one of them has repeated measures of airway resistance to study changes in associations with age. Findings of these studies are inconsistent. Higher ambient air pollution exposure early in life was associated with higher peripheral airway resistance from impulse oscillometry (R5-R20) at age 16 in a Swedish birth cohort [11], and with higher R_int_ at age 4 in our PIAMA birth cohort [12]. Living within 50 m of a busy road was associated with a higher airway resistance (R_aw_) in a cross-sectional study of children aged 5–7 years from Eastern and Western Germany [13]. In contrast, no association was found between life-time exposure to air pollution and repeated measures of specific airway resistance (S_raw_) at ages 3, 5, 8, and 11 years in a birth cohort from Manchester [14].

With the present study, we add to the currently limited evidence regarding the association between long-term air pollution exposure and airway resistance with age. We analyzed associations of air pollution exposure with R_int_ at age 8 years and changes in R_int_ between 4 and 8 years within the prospective PIAMA (Prevention and Incidence of Asthma and Mite Allergy) birth cohort study for which we previously reported positive associations between R_int_ at the age of 4 years and annual average exposure to NO_2_, PM_2.5_ and “soot” at the birth address [12].

## Methods

### Study design and study population

Details on the PIAMA birth cohort study have been published elsewhere [15, 16]. In brief, pregnant women were recruited from the general population in 1996–1997 through antenatal clinics in the north, west and center of the Netherlands. Non-allergic pregnant women were invited to participate in a “natural history” study arm. Pregnant women identified as allergic through a screening questionnaire were allocated primarily to an intervention arm with a random subset allocated to the natural history arm. The intervention involved the use of mite-impermeable mattress and pillow covers.

The study started with 3963 newborns. Parents completed questionnaires on demographic factors, risk factors for asthma and respiratory symptoms at birth, at the child’s ages 3 months and 1 year and then annually until the age of 8 years [16]. At the age of 8, all children of allergic mothers and a random sample of children of non-allergic mothers (total *n* = 1680) were invited for an extensive medical examination and 1235 participated. As part of the medical examination, interrupter resistance (R_int_) was successfully measured in 1003 children. We excluded children who had used asthma medication during the 12 h prior to the R_int_ testing (*n* = 11) and children with missing data on use of asthma medication (*n* = 9). The final study population for this study consisted of 983 children with successful R_int_ measurements and information on air pollution exposure at the birth address (*n* = 975) and/or current home address (*n* = 965).

The Institutional Review Boards of the participating institutes approved the study protocol, and written informed consent was obtained from the parents or legal guardians of all participants.

### R_int_ measurements

R_int_ at the age of 8 years was our primary outcome. Between October 11, 2004 and December 10, 2005 we measured R_int_ (MicroRint, Micro Medical Ltd., Rochester, Kent, UK) by trained personnel while sitting upright, breathing quietly and wearing a nose clip with support of cheeks and chin [8, 17]. All measurements were performed with a filter (Micro Medical Ltd) in place. Shutter closure was programmed at maximal expiratory tidal flow. R_int_ was calculated as the ratio of mouth pressure before and immediately after occlusion of the airway to airflow (kPa·s·L^− 1^). Tracings were inspected immediately after the measurement in the presence of the child. Rejection criteria were: tachypnea, usage of the vocal cords, extreme neck flexion or extension, and leakage of the mouthpiece. R_int_ was calculated as the median of at least five acceptable measurements out of ten for each child.

For a subset of the participants with R_int_ measurements at age 8, R_int_ measurements from an earlier medical examination at age 4 years, using the same methodology, were available together with information on annual average air pollution exposure at the home address at the time of the 4-year R_int_ measurement (*n* = 521). For these participants we calculated the change in R_int_ between 4 and 8 years of age as a secondary outcome.

### Air pollution exposure assessment

We estimated annual average air pollution concentrations at the participants’ birth addresses and current addresses at the time of the R_int_ measurements with spatial land-use regression models that have been developed within the EU-funded ESCAPE (European Study of Cohorts for Air Pollution Effects) project [18, 19]. These land-use regression models are different from the land-use regression models from the TRAPCA (Traffic-Related Air Pollution and  Childhood Asthma) project [20] that have been used in the earlier analyses at age 4 [12]. The new ESCAPE models have a better performance than the TRAPCA models and enable us to investigate associations with nitrogen oxides (NO_x_) and particulate matter with diameters of less than less than 10 μm (PM_10_) and 2.5–10 μm (PM_coarse_) in addition to nitrogen dioxide (NO_2_), particulate matter with diameters of less than 2.5 μm (PM_2.5_), and PM_2.5_ absorbance (“soot”, determined as the reflectance of PM_2.5_ filters). In brief, for the ESCAPE land-use regression models air pollution monitoring campaigns were performed between October 2008 and February 2010 in the study area. Three 2-week measurements of NO_2_ and NO_x_ were performed at 80 sites within 1 year. Simultaneous measurements of PM_2.5_, PM_10_, PM_coarse_, and PM_2.5_ absorbance were performed at 40 of these sites. Results from the three measurements were averaged to estimate the annual average [21]. We evaluated predictor variables of nearby traffic, population and household density, and land use derived from Geographic Information Systems to explain spatial variation in annual average concentrations. The land-use regression models were then used to estimate annual average air pollution concentrations at participants’ home addresses, for which the same Geographic Information Systems predictor variables were obtained, without adjustment for long-term changes in air pollution levels. Overall model performance was evaluated by leave-one-out cross-validation: Each site was sequentially left out from the model while the included variables were left unchanged. A brief description of the models including their performance is provided in Additional file 1: Table S1. The estimated annual average air pollution concentrations from the land-use regression models were our primary estimates of exposure. Since air pollution measurements were performed in 2008–2010, but cohort participants were born in 1996–1997, in addition, we extrapolated predicted concentrations for the birth addresses (for which the time difference with the ESCAPE measurements was largest) back in time to account for long-term changes in air pollution levels using the ratio between the years prior and after birth and the ESCAPE monitoring year, based on data from routine background monitoring network sites in the study areas (for details see http://www.escapeproject.eu/manuals/). We used data from two years to avoid back-extrapolation being influenced too much by specific weather circumstances in a specific year. This may become important when a cohort was recruited in multiple years.

### Covariates

Covariates were selected *a priori *based on previous analyses at age 4 and published literature. Information on sex, parental education (low: primary school, lower vocational or lower secondary education; medium: intermediate vocational education or intermediate/higher secondary education; high: higher vocational education and university), parental allergy (yes/no), maternal smoking during pregnancy (yes/no), smoking in the child’s home (yes/no), mold or dampness in the living room and/or child’s bedroom (yes/no), any pets in the child’s home (yes/no), use of gas for cooking (yes/no), presence of an unvented gas water heater in the child’s home (yes/no), presence of older siblings (yes/no), and Dutch nationality (yes/no) was obtained from the parent-completed questionnaires. Information on season, participant’s age, height, and weight was collected during the medical examination. Data on ambient temperature and relative humidity on the day of the R_int_ measurements was retrieved from the Royal Netherlands Meteorological Institute (KNMI, http://www.knmi.nl/nederland-nu/klimatologie/gemeten-reeksen). Daily average concentrations of NO_2_, PM_10_, and black smoke (“soot”) on the day of the medical examination were obtained from the Dutch National Air Quality Monitoring Network (NAQMN, https://www.lml.rivm.nl/gevalideerd/index.php/).

### Data analysis

The association of R_int_ at age 8 years with annual average air pollution concentrations at the birth address and current home address at the time of the 8-year R_int_ measurements were analyzed by multiple linear regression with and without adjustment for the potential confounding variables described above. We adjusted for the same potential confounders as in previous analyses at age 4 (i.e. sex, parental education, parental allergy, maternal smoking during pregnancy, smoking in the child’s home, mold or dampness, pets, use of gas for cooking, presence of a unvented gas water heater, older siblings, Dutch nationality) and air pollution levels on the day of the R_int_ measurements. Covariates were selected from the questionnaire that coincided best with the exposure period. We performed available case analyses, which results in slightly different numbers of observations for the different models.

In our secondary analysis, associations of changes in R_int_ from age 4 to 8 years with annual average air pollution concentrations during the period between the two R_int_ measurements, taking into account changes in residential address and occupancy at different addresses, were analyzed by multiple linear regression with and without adjustment for the same confounders (*n* = 519 of the 521 participants had complete information on exposure during that period).

We performed a sensitivity analysis to explore to what extent associations with air pollution exposures at the birth address depended on the use of a purely spatial (ESCAPE non back-extrapolated) or temporal-spatial (ESCAPE back-extrapolated) model or the choice of the land-use regression models (ESCAPE models vs TRAPCA models that were used in analyses with R_int_ at age 4). Moreover, we performed separate analyses for children with and without asthma at age 8 and for children who did and did not change address at any time between birth and the 8-year R_int_ measurement. Asthma was defined as a positive answer to at least two of the three following questions: (1) “Has a doctor ever diagnosed asthma in your child?”, (2) “Has your child had wheezing or whistling in the chest in the last 12 months?”, (3) “Has your child been prescribed asthma medication during the last 12 months?”, a definitions that has been developed by a panel of experts within the MeDALL consortium [22].

Functional relationships of the associations between annual average air pollution concentrations and R_int_ at age 8 were explored using smoothing splines. As exposure-response did not deviate significantly (*p* < 0.05) from linearity, except for PM_2.5_ (see Additional file 1: Figures S1 and S2), air pollution levels were entered as continuous variables without transformation in all models. Residual plots were used to check model assumptions. Associations were assessed in single-pollutant models and are presented as mean change in the dependent variable for an interquartile range increase in exposure to facilitate comparison of effect sizes between pollutants. Statistical significance was defined by a two-sided α-level ≤ 5%, marginal statistical significance by a two-sided α-level ≤ 10%.

All analyses were performed using SAS statistical software (version 9.4; SAS Institute Cary, NC, USA).

## Results

Characteristics of the study population are presented in Table 1. About half of the participants were female and most had a Dutch nationality. By design, participants of the intervention study were overrepresented among participants of the 8-year medical examination and consequently the percentage of children with allergic parents was higher in the current study population than in the full cohort (75% vs 51%). Other than that, differences between the current study population and the full cohort were small (see Additional file 1: Table S2). Mean (SD) R_int_ at age 8 was 0.66 (0.16) kPa·s·L^− 1^ (Table 2), which is slightly higher than what would be expected based on published reference values for children being about 1.30 m tall [23]. R_int_ at age 8 years was on average (SD) 0.30 (0.21) kPa·s·L^− 1^ lower than R_int_ at age 4 years.

**Table 1:** Description of the study population

Variable	n/N	(%)
Female sex	504/983	(51)
Parental education		
Low	110/981	(11)
Medium	343/981	(35)
High	528/981	(54)
Parental allergy	739/983	(75)
Maternal smoking during pregnancy	147/974	(15)
Smoking in the child’s home		
First year of life	233/980	(24)
Current^a^	138/916	(15)
Mold/dampness in living room and/or child’s bedroom		
First year of life	69/970	(7)
Current^a^	51/910	(6)
Pets in the child’s home		
First year of life	446/981	(45)
Current^a^	454/900	(50)
Use of gas for cooking		
First year of life	788/965	(82)
Current^a^	732/958	(76)
Unvented gas water heater		
First year of life	44/931	(5)
Current^a^	21/932	(2)
Older siblings	469/982	(48)
Dutch nationality	913/964	(95)
Asthma^b^	103/957	(11)
Did not move house since birth	505/973	(52)

^a^Age 8 years except for use of gas for cooking and unvented gas water heater, where no information was available from the 8-year questionnaire and data from the 5-year questionnaire were used

^b^Defined as 2 out of the 3 following criteria: asthma ever, wheeze in the past 12 months and prescription of asthma medication in the past 12 months

**Table 2:** Description of R_int_ measurements at age 8 years

Variable	N	Mean (SD)
Rint [kPa·s·L^−1^]	983	0.66 (0.16)
Age^a^ [years]	983	8.1 (0.3)
Height^a^ [cm]	983	132.8 (5.7)
Weight^a^ [kg]	983	28.9 (4.9)

^a^At the time of R_int_ measurements

The distributions of the estimated annual average air pollution concentrations at the participants’ birth address, current home address at the time of the 8-year R_int_ measurements, and for the period between the 4- and 8-year R_in__t_ measurements are shown in Table 3. Exposure contrasts were larger for NO_2_, NO_x_ and PM_2.5_ absorbance than for PM_2.5_, PM_10_ and PM_coarse_. Distributions of daily average air pollution concentrations, temperature and relative humidity on the day of the R_int_ measurements are presented in Table S3 in Additional file 1. Correlations between annual average concentrations of NO_2_, NO_x_ and PM_2.5_ absorbance were high for both birth and current addresses (*r* = 0.90–0.92, see Additional file 1: Table S4) and moderate to high for PM_10_ and PM_coarse_. Correlations between annual average concentrations at the birth address and current address for the same pollutant were high (*r* = 0.74–0.85). Correlations between annual average air pollution concentrations and daily average concentrations on the day of the R_int_ measurements were generally low (*r* = 0.02–0.42, see Additional file 1: Table S5).

**Table 3:** Distribution of annual average air pollution concentrations at the participants’ birth address and current home address, and for the period between the 4- and 8-year R_int_ measurements

	Min	P25	Median	Mean	P75	Max	N
Annual average birth address							
NO_2_ [μg/m^3^]	9.4	18.9	23.3	23.4	27.3	48.1	975
NO_x_ [μg/m^3^]	16.5	27.6	33.5	34.8	38.8	88.9	975
PM_2.5_ [μg/m^3^]	15.3	15.7	16.5	16.4	16.8	21.1	975
PM_10_ [μg/m^3^]	23.7	24.1	24.7	25.0	25.4	33.2	975
PM_coarse_ [μg/m^3^]	7.6	7.8	8.1	8.4	8.7	13.0	975
PM_2.5_ absorbance [10^− 5^/m]	0.85	1.09	1.24	1.25	1.36	2.99	975
Annual average at current address^a^							
NO_2_ [μg/m^3^]	9.4	18.2	22.7	22.6	26.6	52.1	965
NO_x_ [μg/m^3^]	16.5	26.1	32.1	33.3	37.3	100.1	965
PM_2.5_ [μg/m^3^]	14.9	15.6	16.5	16.4	16.8	19.3	965
PM_10_ [μg/m^3^]	23.7	24.0	24.6	24.8	25.2	29.8	965
PM_coarse_ [μg/m^3^]	7.6	7.8	8.0	8.3	8.5	11.2	965
PM_2.5_ absorbance [10^−5^/m]	0.85	1.06	1.22	1.22	1.33	2.13	965
Annual average for the period between the 4- and 8-year Rint measurement^b^							
NO_2_ [μg/m^3^]	9.4	19.1	23.0	22.9	26.7	40.4	519
NO_x_ [μg/m^3^]	16.5	27.0	32.8	33.9	37.8	82.7	519
PM_2.5_ [μg/m^3^]	15.3	15.8	16.5	16.4	16.8	20.4	519
PM_10_ [μg/m^3^]	23.7	24.1	24.6	24.9	25.2	33.3	519
PM_coarse_ [μg/m^3^]	7.6	7.8	8.1	8.3	8.5	11.9	519
PM_2.5_ absorbance [10^−5^/m]	0.85	1.09	1.24	1.23	1.33	1.99	519

^a^At the time of the 8-year R_int_ measurement

^b^only for participants with successful R_int_ measurements at both, ages 4 and 8 years

R_int_ tended to be higher in children with higher estimated annual average concentrations of all pollutants except PM_coarse_ at the current address, but this was less consistent for exposures at the birth address (Table 4). Associations attenuated after adjustment for potential confounders, but remained marginally statistically significant for NO_2_, NO_x_ and PM_2.5_ absorbance at the current address and PM_2.5_ at the birth address (*p* < 0.10). R_int_ was on average between 0.011 and 0.018 kPa·s·L^− 1^ higher per interquartile range increase in exposure to these pollutants, which corresponds to 2–3% of the average R_int_ of 0.66 kPa·s·L^− 1^.

**Table 4:** Associations^a^ between R_int_ and estimated annual average air pollution concentrations at the birth address and current home address from single-pollutant models

Pollutant [increment]	Model 1^b^			Model 2^c^		
	β	(95% CI)	*p*-value	β	(95% CI)	*p*-value
Birth address		*N* = 975			*N* = 869	
NO_2_ [8.4 μg/m^3^]	0.011	(− 0.001, 0.024)	0.0831	0.005	(− 0.010, 0.021)	0.4770
NO_x_ [11.2 μg/m^3^]	0.007	(− 0.002, 0.017)	0.1195	0.002	(−0.008, 0.013)	0.6868
PM_2.5_ [1.1 μg/m^3^]	0.023	(0.007, 0.039)	0.0056	0.017	(−0.001, 0.034)	0.0611
PM_10_ [1.3 μg/m^3^]	0.006	(−0.004, 0.016)	0.2612	0.002	(−0.008, 0.012)	0.7013
PM_coarse_ [0.9 μg/m^3^]	0.003	(−0.008, 0.013)	0.6354	0.002	(−0.007, 0.010)	0.7391
PM_2.5_ abs. [0.27 10^−5^/m]	0.013	(0.001, 0.024)	0.0274	0.008	(−0.004, 0.021)	0.1813
Current address^d^		*N* = 965			*N* = 808	
NO_2_ [8.4 μg/m^3^]	0.022	(0.008, 0.035)	0.0016	0.018	(0.001, 0.035)	0.0334
NO_x_ [11.2 μg/m^3^]	0.016	(0.006, 0.026)	0.0025	0.011	(−0.001, 0.022)	0.0781
PM_2.5_ [1.1 μg/m^3^]	0.024	(0.006, 0.042)	0.0106	0.017	(−0.004, 0.039)	0.1079
PM_10_ [1.1 μg/m^3^]	0.012	(0.001, 0.023)	0.0285	0.005	(−0.007, 0.017)	0.4275
PM_coarse_ [0.7 μg/m^3^]	0.006	(−0.004, 0.016)	0.2335	0.000	(−0.011, 0.011)	0.9974
PM_2.5_ abs. [0.27 10^−5^/m]	0.020	(0.007, 0.032)	0.0017	0.014	(0.000, 0.029)	0.0496

^a^Associations are presented as mean difference in R_int_ per interquartile range increase in air pollution exposure (β) with 95% confidence intervals (CI)

^b^Adjusted for sex and age

^c^Adjusted for sex, age, height, weight, parental education, parental allergies, maternal smoking during pregnancy, smoking in the child’s home, mold/dampness in living room and/or child’s bedroom, pets in the child’s home, use of gas for cooking, unvented gas water heater, older siblings, Dutch nationality, season; average air pollution concentration (NO_2_ in models with long-term NO_2_ and NO_x_; PM_10_ in models with long-term PM_2.5_, PM_10_, and PM_coarse_; black smoke in models with long-term PM_2.5_ absorbance), ambient temperature and relative humidity on the day of the R_int_ test

^d^At the time of the Rint measurements

Annual average air pollution exposure at during the period between the 4- and 8-year R_int_ measurements was not associated with the change in R_int_ between 4 and 8 years (see Additional file 1: Table S6).

**Fig. 1:**
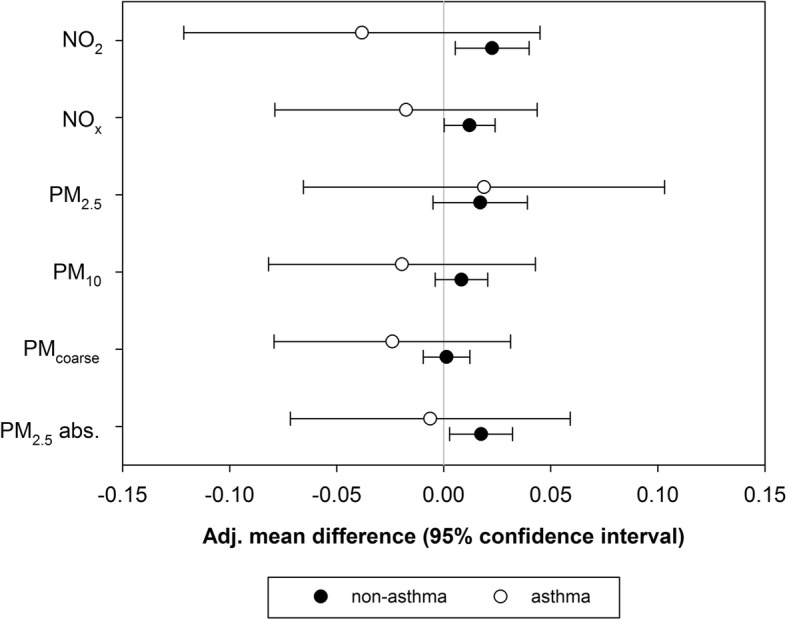
Associations between R_int_ and estimated annual average concentrations at the current home address for children with and without asthma at age 8 years. Associations are presented as mean difference in R_int_ per interquartile range increase in air pollution exposure (β) with 95% confidence intervals (CI). Adjusted for sex, age, height, weight, parental education, parental allergies, maternal smoking during pregnancy, smoking in the child’s home, mold/dampness in living room and/or child’s bedroom, pets in the child’s home, use of gas for cooking, unvented gas water heater, older siblings, Dutch nationality, season; average air pollution concentration (NO_2_ in models with long-term NO_2_ and NO_x_; PM_10_ in models with long-term PM_2.5_, PM_10_, and PM_coarse_; black smoke in models with long-term PM_2.5_ absorbance), ambient temperature and relative humidity on the day of the R_int_ test

**Fig. 2:**
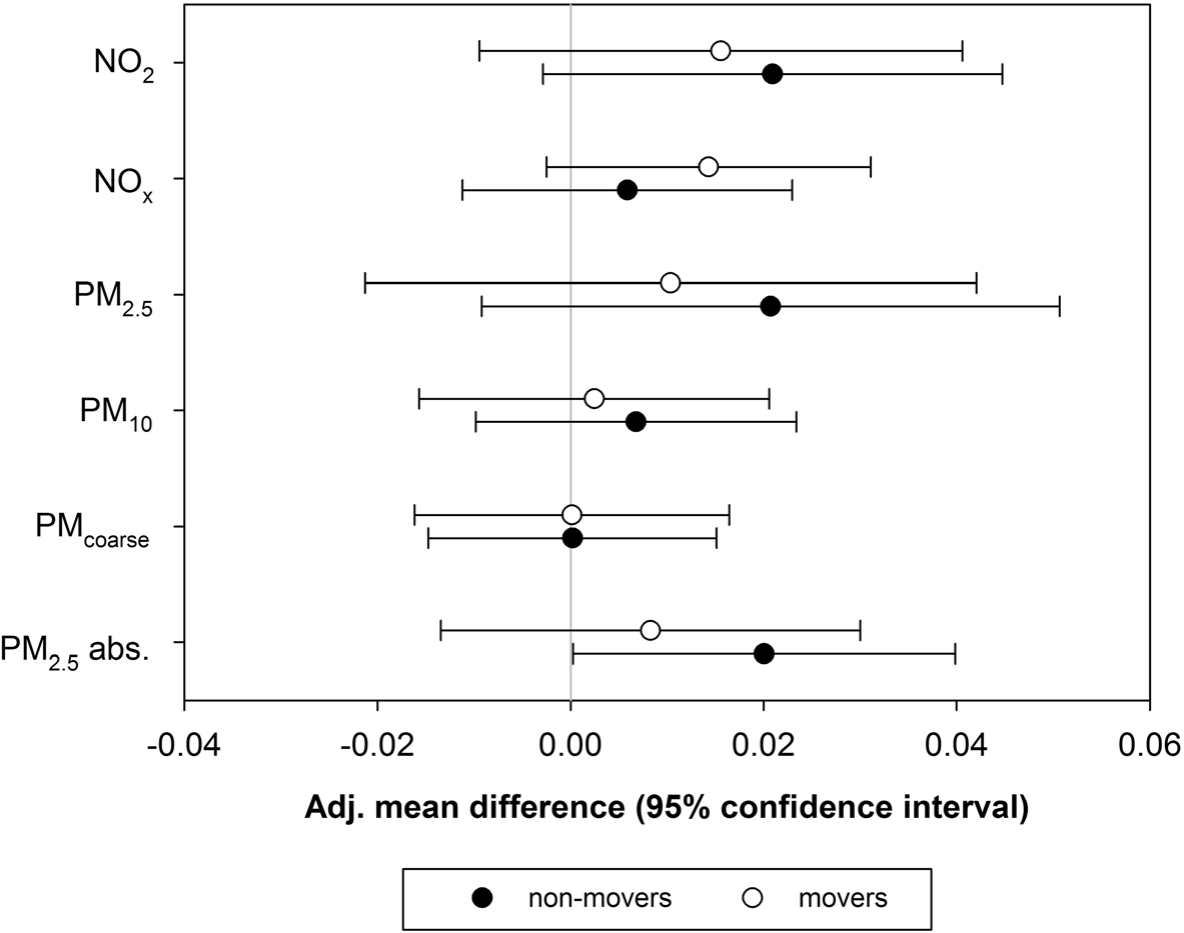
Associations between R_int_ and estimated annual average concentrations at the current home address for children who did and who did not change address at any time between birth and the R_int_ measurements. Associations are presented as mean difference in R_int_ per interquartile range increase in air pollution exposure (β) with 95% confidence intervals (CI). Adjusted for sex, age, height, weight, parental education, parental allergies, maternal smoking during pregnancy, smoking in the child’s home, mold/dampness in living room and/or child’s bedroom, pets in the child’s home, use of gas for cooking, unvented gas water heater, older siblings, Dutch nationality, season; average air pollution concentration (NO_2_ in models with long-term NO_2_ and NO_x_; PM_10_ in models with long-term PM_2.5_, PM_10_, and PM_coarse_; black smoke in models with long-term PM_2.5_ absorbance), ambient temperature and relative humidity on the day of the R_int_ test

Adjusted associations of R_int_ at age 8 with air pollution concentrations were very similar for back-extrapolated ESCAPE models and the older TRAPCA land-use regression models for NO_2_, PM_2.5_, and PM_2.5_ absorbance that we used in previous analyses at age 4 instead of the more recent ESCAPE land-use regression models that were used in the main analysis, but twice as big as associations with non back-extrapolated exposure estimates from the ESCAPE models (Table 5). Associations with exposure at the current address were limited to non-asthmatics (Fig. 1), but the number of asthmatics was small and consequently confidence intervals were wide. The associations with annual average exposure at the current address did not differ between participants who did and who did not change address at any time between birth and the R_int_ measurements (Fig. 2).

**Table 5:** Adjusted associations^a^ between R_int_ and estimated annual average concentrations at the birth address from single-pollutant models – ESCAPE non back-extrapolated vs ESCAPE back-extrapolated vs TRAPCA land-use regression models

Pollutant	ESCAPE model – non back-extrapolated			ESCAPE model –back-extrapolated			TRAPCA model		
	β	(95% CI)	*p*-value	β	(95% CI)	*p*-value	β	(95% CI)	*p*-value
NO_2_	0.005	(−0.010, 0.021)	0.4770	0.012	(−0.001, 0.026)	0.0768	0.011	(−0.003, 0.024)	0.1228
NO_x_	0.002	(−0.008, 0.013)	0.6868	0.009	(−0.001, 0.019)	0.0935	---^b^		
PM_2.5_	0.017	(−0.001, 0.034)	0.0611	0.026	(0.009, 0.043)	0.0022	0.012	(−0.005, 0.029)	0.1543
PM_10_	0.002	(−0.008, 0.012)	0.7013	0.017	(0.001, 0.033)	0.0340	---^b^		
PM_coarse_	0.002	(−0.007, 0.010)	0.7391	0.010	(−0.002, 0.023)	0.1144	---^b^		
PM_2.5_ abs.	0.008	(−0.004, 0.021)	0.1813	0.016	(0.004, 0.029)	0.0113	0.012^b^	(−0.002, 0.026)	0.1055

^a^Associations are presented as mean difference in R_int_ per interquartile range increase in air pollution exposure (β) with 95% confidence intervals (CI). Adjusted for sex, age, height, weight, parental education, parental allergies, maternal smoking during pregnancy, smoking in the child’s home, mold/dampness in living room and/or child’s bedroom, pets in the child’s home, use of gas for cooking, unvented gas water heater, older siblings, Dutch nationality, season; average air pollution concentration (NO_2_ in models with long-term NO_2_ and NO_x_; PM_10_ in models with long-term PM_2.5_, PM_10_, and PM_coarse_; black smoke in models with long-term PM_2.5_ absorbance), ambient temperature and relative humidity on the day of the R_int_ test

^b^Not available

## Discussion

Our results provide evidence that R_int_ at age 8 years was higher in children with higher estimated annual average air pollution concentrations, in particular in children with higher concentrations of NO_2_, NO_x_ and PM_2.5_ absorbance at the current address.

Our findings contribute to the growing body of evidence on the long-term effects of air pollution exposure on children’s lung function. Most studies performed so far linked air pollution exposure to spirometry data [5] and FEV_1_ mostly reflects large airway patency [24]. The R_int_ technique that has been used in the present study has been shown to detect changes in proximal and more distal airway function [25]. Given the low correlation (r = -0.41) between R_int_ and FEV_1_ at age 8 in our study population, the present analyses may provide additional insight into the adverse effects of air pollution on the airways of children.

The present analysis extends earlier analyses of associations between air pollution exposure at the birth address and R_int_ at age 4 years in the same cohort [12]. Height has been found to be the best predictor of R_int_ in children and the observed decrease from age 4 to age 8 is in accordance with published reference eqs. [23]. The observed 2–3% higher R_int_ at age 8 years per interquartile range increase in air pollution levels is consistent with the association estimates at age 4 years (i.e. 0.025–0.031 kPa·s·L^− 1^ per interquartile range increase in exposure, which corresponds to 3% of the mean R_int_ of 0.96 kPa·s·L^− 1^). Together with our finding that there was no association between air pollution exposure and change in R_int_ between ages 4 and 8 years this suggests that the difference in R_int_ between participants with high and low levels of air pollution exposure does not further increase with age, but remains rather constant between ages 4 and 8 years. Few other studies assessed the association between air pollution and airway resistance. Our findings confirm the findings of a Swedish birth cohort study that found that higher levels of NO_x_ and PM_10_ early in life were associated with higher peripheral airway resistance (R5-R20) at age 16 [11]. In a cross-sectional study of more than 2500 children aged 5–7 years from Eastern and Western Germany living within 50 m of a busy road, but not annual average concentration of total suspended particles, was associated with higher R_aw_ [13]. In contrast, no associations were found between life-time exposure to nitrogen dioxide (NO_2_) and particulate matter with a diameter of less than 10 μm (PM_10_) and repeated measures of S_raw_ at ages 3, 5, 8, and 11 years in a birth cohort from Manchester [14]. A quantitative comparison of the observed air pollution effects between our study and the other studies is limited by the different outcomes and exposure measures that were used.

An advantage of the current analysis over the earlier analyses at age 4 is that we were able to investigate the relevance of early life versus recent exposure. The more consistent associations with exposure at the current address as compared to exposure at the birth address are consistent with findings for FEV_1_ and FVC at age 6–8 years from five European birth cohorts including PIAMA [26]. Further evidence for an association of airway resistance with current air pollution exposure comes from the German study [13]. So far, only the Swedish study has assessed associations of airway resistance with air pollution exposure at different time points and found, opposite to the present study, associations with exposure during the first year, but not during the year preceding the lung function measurements [11].

Oxidative stress-induced inflammation has been hypothesized as a main mechanism underlying the respiratory health effects of air pollution [6]. We observed associations with airway resistance in particular for the more traffic-related pollutants NO_2_, NO_x_ and PM_2.5_ absorbance and less consistently with particulate matter mass concentrations (PM_2.5_, PM_10_, and PM_coarse_). However, the relevance of specific air pollutants remains unclear due to the high spatial correlation between pollutants, which is an inherent limitation of population studies investigating air pollution effects under real life conditions. Also, the more consistent associations with NO_2_, NO_x_ and PM_2.5_ absorbance could be at least partly explained by the better performance of the land-use regression models for NO_2_, NO_x_ and PM_2.5_ absorbance as compared to the PM models (see Additional file 1: Table S1) and consequently a smaller exposure measurement error for these pollutants. Since R_int_ measurements are probably influenced by the resistance of small airways, we speculate that an effect of nitrogen oxides and small particles may be due to penetration into small airways.

It can be argued that a potential limitation of our study is that the land-use regression models that we used to estimate exposures were based on measurements performed in 2008–2010, while study participants were born in 1996/97 and airway resistance measurements at age 8 were performed in 2004/2005. However, several studies from Europe and North America have demonstrated that spatial contrasts of air pollutants, in particular NO_2_ and elemental carbon are stable over periods of 7 and more years [27–29]. Moreover, air pollution measurements performed in 2008–2010 were highly correlated with air pollution measurements in 1999–2000 [30]. However, associations of R_int_ at age 8 with exposure at the birth address were about doubled when we used back-extrapolated exposures and estimated exposures from an older land-use regression model that was based on the 1999–2000 measurements suggesting that using non back-extrapolated ESCAPE exposure estimates most likely results in an underestimation of associations with R_int_.

Another potential limitation of our study is that we restricted our study to air pollution exposure at the residential address and did not include non-residential exposures (e.g. at school) and time-activity patterns. Although data from our cohort and the Swedish study show high correlations between home and school address exposures during the primary school period [31, 32], we cannot rule out that measurement error is differential, e.g. that it differs between asthmatic and non-asthmatic children, because of asthmatic children possibly being more likely to spend more time at home.

Children with at least one allergic parent were overrepresented in our study sample (75% vs 51% in the full PIAMA cohort). Together with the fact that highly educated Dutch parents are over-represented in the PIAMA cohort, this may limit the generalizability of our findings to the full PIAMA cohort and to the general population.

## Conclusions

In conclusion, our results support the hypothesis that air pollution exposure is associated with a higher airway resistance in schoolchildren.

## Additional file

Additional file 1: **Table S1.** Land-use regression models with model performance (leave-one-out cross-validation R^2^, R^2^_LOOCV_), **Table S2.** Comparison of characteristics between the study population (*n* = 983) and the full PIAMA cohort (*n* = 3963). **Table S3.** Distribution of daily average air pollution concentrations, temperature and relative humidity on the day of the R_int_ measurements. **Table S4.** Spearman correlations between annual average air pollution concentrations at the participants’ birth and current addresses. **Table S5.** Correlations of estimated annual average air pollution concentrations at the birth and current address at the time of the 8-year R_int_ measurements with daily average air pollution concentrations on the day of the R_int_ tests. **Table S6.** Associations ^*^ between change in R_int_ from age 4 to age 8 years (R_int_ age 4 – R_int_ age 8) and estimated average air pollution concentrations during the period between the two R_int_ measurement from single-pollutant models. **Figure S1.** Smoothing splines of the relationship between annual average air pollution concentrations at the birth address and R_int_ at age 8 from single-pollutant models. **Figure S2.** Smoothing splines of the relationship between annual average air pollution concentrations at the current address at the time of the R_int_ measurement and R_int_ at age 8 from single-pollutant models. (DOCX 1123 kb)

## Abbreviations

ESCAPE: European Study of Cohorts for Air Pollution Effects
FEV_1_: Forced expiratory volume within 1 s
NO_2_: Nitrogen dioxide
NO_x_: Nitrogen oxides
PIAMA: Prevention and Incidence of Asthma and Mite Allergy
PM_10_: Particulate matter with a diameter of less than 10 μm
PM_2.5_: Particulate matter with a diameter of less than 2.5 μm
PM_coarse_: Particulate matter with a diameter 2.5–10 μm
R_aw_: Airway resistance
R_int_: Interrupter resistance
TRAPCA: Traffic-Related Air Pollution and Childhood Asthma
