# Supplementary material for: Air pollution and airway resistance at age 8 years – the PIAMA birth cohort study
Source: Environ Health. 2018 Jul 17;17:61. doi: 10.1186/s12940-018-0407-9 (PMC6050657; doi:10.1186/s12940-018-0407-9)
Supplement: Supplementary file 1 — Table S1. Land-use regression models with model performance (leave-one-out cross-validation R2, R2LOOCV), Table S2. Comparison of characteristics between the study population (n = 983) and the full PIAMA cohort (n = 3963). Table S3. Distribution of daily average air pollution concentrations, temperature and relative humidity on the day of the Rint measurements. Table S4. Spearman correlations between annual average air pollution concentrations at the participants’ birth and current addresses. Table S5. Correlations of estimated annual average air pollution concentrations at the birth and current address at the time of the 8-year Rint measurements with daily average air pollution concentrations on the day of the Rint tests. Table S6. Associations * between change in Rint from age 4 to age 8 years (Rint age 4 – Rint age 8) and estimated average air pollution concentrations during the period between the two Rint measurement from single-pollutant models. Figure S1. Smoothing splines of the relationship between annual average air pollution concentrations at the birth address and Rint at age 8 from single-pollutant models. Figure S2. Smoothing splines of the relationship between annual average air pollution concentrations at the current address at the time of the Rint measurement and Rint at age 8 from single-pollutant models. (DOCX 1123 kb) [file 12940_2018_407_MOESM1_ESM.docx]

**Air pollution and airway resistance at age 8 years – the PIAMA birth cohort study**

Isabelle Finke, Henriette A. Smit, Alet H. Wijga, Gerard H. Koppelman, Judith Vonk, Bert Brunekreef, Ulrike Gehring

**Supplemental Material**

**Table S1.** Land-use regression models with model performance (leave-one-out cross-validation R^2^, R^2^_LOOCV_)

| **Exposure** | **Land-use regression model** | **R^2^_LOOCV_** |
| --- | --- | --- |
| NO_2_ | -7.80 + 1.18 × REGIONALESTIMATE + 2.30 × 10^-5^ × POP_5000 + 2.46 × 10^-6^ × TRAFLOAD_50 + 1.06 × 10^-4^ × ROADLENGTH_1000  + 9.84 × 10^-5^ × HEAVYTRAFLOAD_25 +12.19 × DISTINVNEARC1 + 4.47 × 10^-7^ × HEAVYTRAFLOAD_25_500 | 0.81 |
| NO_x_ | 3.25 + 0.74 × REGIONALESTIMATE + 4.22 × 10^-6^× TRAFLOAD_50 + 6.36 × 10^-4^ × POP_1000 + 2.39 × 10^-6^ × HEAVYTRAFLOAD_500  + 71.65 × DISTINVMAJOR1 + 0.21 × MAJORROADLENGTH_25 | 0.82 |
| PM_2.5_ abs | 0.07 + 2.95 × 10^−9^ × TRAFLOAD_500 + 2.93 × 10^−3^ × MAJORROADLENGTH_50 + 0.85 × REGIONALESTIMATE  + 7.90 × 10^−9^ × HLDRES_5000 + 1.72 × 10^−6^ × HEAVYTRAFLOAD_50 | 0.89 |
| PM_10_ | 23.71 + 2.16 × 10^-8^ × TRAFMAJORLOAD_500 + 6.68 × 10^-6^ × POP_5000 + 0.02 × MAJORROADLENGTH_50 | 0.60 |
| PM_2.5_ | 9.46 + 0.42 × REGIONALESTIMATE + 0.01 × MAJORROADLENGTH_50 + 2.28 × 10^−9^ × TRAFMAJORLOAD_1000 | 0.61 |
| PM_coarse_ | 7.59 + 5.02 × 10^−9^ × TRAFLOAD_1000 + 1.38 × 10^−7^ × PORT_5000 + 5.38 × 10^−5^ × TRAFNEAR | 0.38 |

DISTINVMAJOR1: inverse distance (m^-1^) to the nearest road of the local road network; DISTINVNEARC1: Inverse distance to the nearest road; HEAVYTRAFLOAD_X: Total heavy-duty traffic load of all roads in X m buffer (sum of (heavy-duty traffic intensity *length of all segments)); HLDRES_X: Sum of high density and low density residential land in X m buffer; MAJORROADLENGTH_X: Road length of major roads in X m buffer; POP_X: Number of inhabitants in X m buffer; PORT: port in X m buffer; REGIONALESTIMATE: Regional estimate; ROADLENGTH_X: Road length of major roads in X m buffer; TRAFLOAD_X: Total traffic load of all roads in X m buffer (sum of (traffic intensity * length of all segments)); TRAFMAJORLOAD_X: Total traffic load of major roads in X m buffer (sum of (traffic intensity * length of all segments)); TRAFNEAR: Traffic intensity on nearest road;

**Table S2.** Comparison of characteristics between the study population (n=983) and the full PIAMA cohort (n=3,963).

|  | **Study population** | |  | **Full cohort** | |
| --- | --- | --- | --- | --- | --- |
| **Variable** | **n/N** | **(%)** |  | **n/N** | **(%)** |
| Female sex | 504/983 | (51) |  | 1,909/3,963 | (48) |
| Parental education |  |  |  |  |  |
| Low | 110/981 | (11) |  | 502/3,812 | (13) |
| Medium | 343/981 | (35) |  | 1,402/3,812 | (37) |
| High | 528/981 | (54) |  | 1,908/3,812 | (50) |
| Parental allergy | 739/983 | (75) |  | 2,038/3,963 | (51) |
| Maternal smoking during pregnancy | 147/974 | (15) |  | 700/3,926 | (18) |
| Smoking in the child’s home |  |  |  |  |  |
| First year of life | 233/980 | (24) |  | 1,058/3,815 | (28) |
| Current | 138/916 | (15) |  | 548/3,254 | (17) |
| Mold or dampness in the child’s home |  |  |  |  |  |
| First year of life | 69/970 | (7) |  | 312/3,698 | (8) |
| Current | 51/910 | (6) |  | 214/3,236 | (7) |
| Pets in the child’s home |  |  |  |  |  |
| First year of life | 446/981 | (45) |  | 1,904/3,833 | (50) |
| Current | 454/900 | (50) |  | 1,820/3,209 | (57) |
| Use of gas for cooking |  |  |  |  |  |
| First year of life | 788/965 | (82) |  | 3,028/3,719 | (81) |
| Current | 732/958 | (76) |  | 2,743/3,507 | (78) |
| Unvented gas water heater |  |  |  |  |  |
| First year of life | 44/931 | (5) |  | 159/3,586 | (4) |
| Current | 21/932 | (2) |  | 74/3,392 | (2) |
| Older siblings | 469/982 | (48) |  | 1,989/3,936 | (51) |
| Dutch nationality | 913/964 | (95) |  | 3,485/3,700 | (94) |

**Table S3.** Distribution of daily average air pollution concentrations, temperature and relative humidity on the day of the R_int_ measurements.

|  | **Min** | **P25** | **Median** | **Mean** | **P75** | **Max** | **N** |
| --- | --- | --- | --- | --- | --- | --- | --- |
| **Day of R_int_ measurements** | | | | | | | |
| NO_2_ [µg/m³] | 1.0 | 14.0 | 23.0 | 24.9 | 34.0 | 92.0 | 965 |
| PM_10_ [µg/m³] | 5.0 | 19.0 | 26.0 | 29.0 | 36.0 | 91.0 | 965 |
| Black smoke [µg/m³] | 0.0 | 2.0 | 5.0 | 7.0 | 10.0 | 38.0 | 965 |
| Temperature [^o^C] | -5.5 | 4.0 | 9.0 | 8.7 | 12.4 | 25.6 | 965 |
| Rel. humidity [%] | 51.0 | 76.0 | 85.0 | 82.5 | 89.0 | 100.0 | 965 |

**Table S4.** Spearman correlations between annual average air pollution concentrations at the participants’ birth and current addresses.

|  | **Birth address** | | | | | |  | **Current address ^*^** | | | | | |
| --- | --- | --- | --- | --- | --- | --- | --- | --- | --- | --- | --- | --- | --- |
|  | **NO_2_** | **NO_x_** | **PM_2.5_** | **PM_10_** | **PM_coarse_** | **PM_2.5_ abs.** |  | **NO_2_** | **NO_x_** | **PM_2.5_** | **PM_10_** | **PM_coarse_** | **PM_2.5_ abs.** |
| **Birth address** | |  |  |  |  |  |  |  |  |  |  |  |  |
| NO_2_ | 1.00 | 0.91 | 0.72 | 0.83 | 0.76 | 0.92 |  | 0.85 | 0.76 | 0.62 | 0.67 | 0.61 | 0.79 |
| NO_x_ |  | 1.00 | 0.77 | 0.84 | 0.77 | 0.91 |  | 0.78 | 0.79 | 0.65 | 0.66 | 0.62 | 0.76 |
| PM_2.5_ |  |  | 1.00 | 0.71 | 0.68 | 0.84 |  | 0.64 | 0.66 | 0.81 | 0.56 | 0.53 | 0.71 |
| PM_10_ |  |  |  | 1.00 | 0.82 | 0.91 |  | 0.69 | 0.66 | 0.57 | 0.75 | 0.63 | 0.72 |
| PM_coarse_ |  |  |  |  | 1.00 | 0.75 |  | 0.62 | 0.61 | 0.52 | 0.62 | 0.74 | 0.59 |
| PM_2.5_ abs. |  |  |  |  |  | 1.00 |  | 0.79 | 0.75 | 0.70 | 0.71 | 0.60 | 0.83 |
| **Current address ^*^** | |  |  |  |  |  |  |  |  |  |  |  |  |
| NO_2_ |  |  |  |  |  |  |  | 1.00 | 0.92 | 0.71 | 0.82 | 0.72 | 0.92 |
| NO_x_ |  |  |  |  |  |  |  |  | 1.00 | 0.74 | 0.84 | 0.76 | 0.90 |
| PM_2.5_ |  |  |  |  |  |  |  |  |  | 1.00 | 0.67 | 0.64 | 0.82 |
| PM_10_ |  |  |  |  |  |  |  |  |  |  | 1.00 | 0.79 | 0.90 |
| PM_coarse_ |  |  |  |  |  |  |  |  |  |  |  | 1.00 | 0.71 |
| PM_2.5_ abs. |  |  |  |  |  |  |  |  |  |  |  |  | 1.00 |

^*^ at the time of the R_int_ measurements

**Table S5.** Correlations of estimated annual average air pollution concentrations at the birth and current address at the time of the 8-year R_int_ measurements with daily average air pollution concentrations on the day of the R_int_ tests.

|  | **Day of R_int_ measurements** | | |
| --- | --- | --- | --- |
|  | **NO_2_** | **PM_10_** | **Black smoke** |
| **Birth address** | |  |  |
| NO_2_ | 0.37 | 0.13 | 0.08 |
| NO_x_ | 0.30 | 0.10 | 0.08 |
| PM_2.5_ | 0.29 | 0.08 | 0.14 |
| PM_10_ | 0.24 | 0.10 | 0.05 |
| PM_coarse_ | 0.19 | 0.08 | 0.02 |
| PM_2.5_ abs. | 0.35 | 0.14 | 0.10 |
| **Current address ^*^** | |  |  |
| NO_2_ | 0.42 | 0.16 | 0.10 |
| NO_x_ | 0.35 | 0.12 | 0.10 |
| PM_2.5_ | 0.34 | 0.12 | 0.18 |
| PM_10_ | 0.29 | 0.13 | 0.08 |
| PM_coarse_ | 0.22 | 0.09 | 0.03 |
| PM_2.5_ abs. | 0.41 | 0.17 | 0.13 |

^*^ at the time of the R_int_ measurements

**Table S6.** Associations ^*^ between change in R_int_ from age 4 to age 8 years (R_int_ age 4 – R_int_ age 8) and estimated average air pollution concentrations during the period between the two R_int_ measurement from single-pollutant models

|  | **Model 1** ^†^ **(N = 519)** | | |  | **Model 2** ^‡^ **(N = 413)** | | |
| --- | --- | --- | --- | --- | --- | --- | --- |
| **Pollutant [increment]** | **β** | **(95% CI)** | **p-value** |  | **β** | **(95% CI)** | **p-value** |
| NO_2_ [7.4 µg/m³] | -0.008 | (-0.031 ,0.015 ) | 0.4986 |  | -0.009 | (-0.044 ,0.026 ) | 0.6120 |
| NO_x_ [9.7 µg/m³] | -0.007 | (-0.025 ,0.011 ) | 0.4667 |  | -0.013 | (-0.034 ,0.009 ) | 0.2565 |
| PM_2.5_ [0.9 µg/m³] | 0.006 | (-0.022 ,0.034 ) | 0.6704 |  | 0.008 | (-0.025 ,0.042 ) | 0.6319 |
| PM_10_ [1.1 µg/m³] | -0.009 | (-0.026 ,0.009 ) | 0.3420 |  | -0.008 | (-0.030 ,0.013 ) | 0.4529 |
| PM_coarse_ [0.8 µg/m³] | 0.005 | (-0.013 ,0.023 ) | 0.5909 |  | 0.009 | (-0.013 ,0.031 ) | 0.4341 |
| PM_2.5_ abs. [0.24 10^-5^/m] | -0.008 | (-0.028 ,0.012 ) | 0.4531 |  | -0.015 | (-0.042 ,0.011 ) | 0.2561 |

^*^ Associations are presented as mean difference in R_int_ per interquartile range increase in air pollution exposure (β) with 95% confidence intervals (CI).

^†^ Adjusted for sex, age at the 4-year R_int_ measurement and difference in age between the 4 – and 8-year measurements

^‡^ Adjusted for sex, age, height, weight, parental education, parental allergies, maternal smoking during pregnancy, smoking in the child’s home, mold/dampness in living room and/or child’s bedroom, pets in the child’s home, use of gas for cooking, unvented gas water heater, older siblings, Dutch nationality, season; average air pollution concentration (NO_2_ in models with long-term NO_2_ and NO_x_; PM_10_ in models with long-term PM_2.5_, PM_10_, and PM_coarse_; black smoke in models with long-term PM_2.5_ absorbance), ambient temperature and relative humidity on the day of the R_int_ test. For age, height and weight, both, values at the time of the 4-year measurements and differences between 4- and 8-year measurements were included.

**Figure S1.** Smoothing splines of the relationship between annual average air pollution concentrations at the birth address and R_int_ at age 8 from single-pollutant models.

**Figure S2.** Smoothing splines of the relationship between annual average air pollution concentrations at the current address at the time of the R_int_ measurement and R_int_ at age 8 from single-pollutant models.
